# Supplementary material for: Attitudes and Knowledge of European Medical Students and Early Graduates about Vaccination and Self-Reported Vaccination Coverage—Multinational Cross-Sectional Survey
Source: Int J Environ Res Public Health. 2021 Mar 30;18(7):3595. doi: 10.3390/ijerph18073595 (PMC8036942; doi:10.3390/ijerph18073595)
Supplement: Supplementary file 1 [file ijerph-18-03595-s001.zip › 1. Questionnaire.docx]

**QUESTIONNAIRE**

**GENERAL INFORMATION:**

**What is your gender?** (choose: male / female / other) **What year you were born in?** (text box)

**In which country are you enrolled?** (text box)

**What are you studying?** (text box)

**Which year of study are you in?** (text box)

*************************************************************************************************************

**Q1: Which statement describes your opinion about vaccinations the most?**

| 1. It is useful and safe and I think that everybody should get vaccinated. |
| --- |
| 1. There is too little evidence to prove that it is effective. |
| 1. There is too little evidence to prove that it is even safe to get vaccinated and I think that nobody should do this. |

**Q2: Do you think that vaccination programs are an effective tool in disease prevention?**

| 1. Yes, I think it is effective. |
| --- |
| 1. I don’t think that it makes a difference because I would choose to vaccinate either way. |
| 1. No because there is not enough proof that vaccines are effective or even safe. |
| 1. No because I don't think that such things should be forced on. |

**Q3: What influences your opinion about vaccinations the most?**

| 1. Scientfic facts |
| --- |
| 1. Social Media |
| 1. Senior physicians, professors |
| 1. My relatives |
| 1. Religious beliefs |
| 1. My friends, colleagues |

**Q4: Did you get vaccinated as part of the vaccination program of your country?**

| 1. Yes |
| --- |
| 1. No |
| 1. I don't know |

**Q5-23:**

| **Which vaccinations have you received as part of the vaccination program:** | Yes | No | I don’t know |
| --- | --- | --- | --- |
| **influenza/seasonal influenza (Q5)** |  |  |  |
| **tetanus (Q6)** |  |  |  |
| **diphtheria (Q7)** |  |  |  |
| **poliomyelitis (Q8)** |  |  |  |
| **Haemophilus influenzae t. B (Q9)** |  |  |  |
| **pertussis (Q10)** |  |  |  |
| **hepatitis A (Q11)** |  |  |  |
| **hepatitis B (Q12)** |  |  |  |
| **pneumococci (Q13)** |  |  |  |
| **measles (Q14)** |  |  |  |
| **mumps (Q15)** |  |  |  |
| **rubella (Q16)** |  |  |  |
| **meningococci (Q17)** |  |  |  |
| **chickenpox (Q18)** |  |  |  |
| **human papillomavirus (HPV) (Q19)** |  |  |  |
| **typhus (Q20)** |  |  |  |
| **cholera (Q21)** |  |  |  |
| **smallpox (Q22)** |  |  |  |
| **tuberculosis (Q23)** |  |  |  |

**Q24: What other vaccinations have you received?**

(text box)

**Q25: Do you know that in order to be protected properly you need to get revaccinated for several vaccines?**

| 1. Yes, I am aware it and doing it properly. |
| --- |
| 1. Yes, I am aware of it, but I am not sure if I have full vaccination. |
| 1. No, this is the first time I hear about that. |
| 1. No, there is no need because vaccination is always life-long protection |

**Q26: Do you advise your relatives, friends, colleagues etc. to get vaccinated?**

| 1. Yes |
| --- |
| 1. No |
| 1. Never thought about that |

**Q27: Do you think that a more specific vaccination program should be available to pregnant women (e.g. seasonal flu, mumps, rubella)?**

| 1. Yes, because that way the fetus will be protected against inborn anomalies and fewer miscarriages will occur |
| --- |
| 1. No, because a specific programme is not safe to a pregnant woman or the fetus |
| 1. No, because the vaccine is not effective for the pregnant woman or the fetus. |
| 1. No, because everyone should have the right to choose. |

**Q28: What is your opinion about the seasonal flu vaccine?**

| 1. It is an almost 100% protection against seasonal flu. |
| --- |
| 1. It is not useful because the seasonal flu virus mutates constantly and there is a different type every year. |
| 1. It won't necessary prevent you from contracting the seasonal flu, but the disease will be less serious. |
| 1. Vaccines in general are not effective and the seasonal flu vaccine is not an exception. |

**Q29: How often do you get vaccinated against seasonal flu?**

| 1. Every other season. |
| --- |
| 1. Every season. |
| 1. I have never been vaccinated against seasonal flu. |
| 1. I haven only been vaccinated once. |
| 1. Not regularly. |

**Q30: Do you think that a vaccine against seasonal flu and hepatitis B should be mandatory for medical staff (attending doctors, nurses etc.)?**

| 1. No, because everyone should have the right to choose. |
| --- |
| 1. No, because those vaccines are not effective. |
| 1. No, because those vaccines are not safe. |
| 1. Yes, because medical staff has a greater chance to get infected and then spread the spread the virus. |

**Q31: Do you think that a vaccine against seasonal flu and hepatitis B should be mandatory for medical students?**

| 1. No, because everyone should have the right to choose. |
| --- |
| 1. No, because these vaccines are not safe. |
| 1. No, because those vaccines are not effective. |
| 1. Yes, because medical students rotate through different departments in a hospital and can spread the virus. |
