# Supplementary material for: Attitudes and Knowledge of European Medical Students and Early Graduates about Vaccination and Self-Reported Vaccination Coverage—Multinational Cross-Sectional Survey
Source: Int J Environ Res Public Health. 2021 Mar 30;18(7):3595. doi: 10.3390/ijerph18073595 (PMC8036942; doi:10.3390/ijerph18073595)
Supplement: Supplementary file 1 [file ijerph-18-03595-s001.zip › 3. Figure and Tables.docx]

**Figure 1*.*** Number of medical students and graduates per country (N=1,821).

**Table 1.** General characteristics of the medical students and junior doctors (N=1,821). JD – junior doctors

| **Characteristics** | **Category** | ***n (%)*** |
| --- | --- | --- |
| Year of birth | up to 1991 | 571 (31.4) |
|  | 1992-1993 | 604 (33.2) |
|  | 1994 or higher | 646 (35.4) |
| Gender | Male | 555 (30.5) |
|  | Female | 1259 (69.1) |
|  | Other | 7 (0.4) |
| Year of study | 1. – 2. | 467 (25.6) |
|  | 3. – 4. | 645 (35.5) |
|  | 5. – 6.* | 651 (35.7) |
|  | JD | 58 (3.2) |

**Table 2.** General attitude towards vaccination and sources of information in Q1-3.

| **Question**  Answer | **Total (N=1821)** | **Gender** | | **Year of study** | | | |
| --- | --- | --- | --- | --- | --- | --- | --- |
|  |  | **Male (N=555)** | **Female (N=1259)** | **1-2 (N=467)** | **3-4 (N=645)** | **5-6 (N=651)** | **JD (N=58)** |
| **Q1. Which statement describes your opinion about vaccinations the most?** | | | | | | | |
| A. It is useful and safe and I think that everybody should get vaccinated. | 1797 (98.7) | 549 (98.9) | 1241 (98.6) | 451 (96.6) | 640 (99.2) | 649 (99.7) | 57 (98.3) |
| B. There is too little evidence to prove that it is effective. | 16 (0.9) | 4 (0.7) | 12 (1.0) | 12 (2.6) | 3 (0.5) | 0 (0.0) | 1 (1.7) |
| C. There is too little evidence to prove that it is even safe to get vaccinated and I think that nobody should do this. | 8 (0.4) | 2 (0.4) | 6 (0.5) | 4 (0.9) | 2 (0.3) | 2 (0.3) | 0 (0.0) |
| **Q2. Do you think that vaccination programs are an effective tool in disease prevention?** | | | | | | | |
| A. Yes, I think it is effective. | 1770 (97.2) | 545 (98.2) | 1219 (96.8) | 438 (93.8) | 630 (97.7) | 645 (99.1) | 57 (98.3) |
| B. I don’t think that it makes a difference because I would choose to vaccinate either way. | 35 (1.9) | 8 (1.4) | 26 (2.1) | 18 (3.9) | 13 (2.0) | 4 (0.6) | 0 (0.0) |
| C. No because there is not enough proof that vaccines are effective or even safe. | 6 (0.3) | 2 (0.4) | 4 (0.3) | 2 (0.4) | 2 (0.3) | 2 (0.3) | 0 (0.0) |
| D. No because I don't think that such things should be forced on. | 10 (0.5) | 0 (0.0) | 10 (0.8) | 9 (1.9) | 0 (0.0) | 0 (0.0) | 1 (1.7) |
| **Q3: What influences your opinion about vaccinations the most?** | | | | | | | |
| A. Scientific facts | 1610 (88.4) | 506 (91.2) | 1099 (87.3) | 388 (83.1) | 573 (88.8) | 593 (91.1) | 56 (96.6) |
| B. Social Media | 19 (1.0) | 4 (0.7) | 15 (1.2) | 9 (1.9) | 3 (0.5) | 7 (1.1) | 0 (0.0) |
| C. Senior physicians, professors | 159 (8.7) | 35 (6.3) | 122 (9.7) | 48 (10.3) | 66 (10.2) | 44 (6.8) | 1 (1.7) |
| D. My relatives | 26 (1.4) | 7 (1.3) | 19 (1.5) | 20 (4.3) | 2 (0.3) | 3 (0.5) | 1 (1.7) |
| E. Religious beliefs | 0 (0.0) | 0 (0.0) | 0 (0.0) | 0 (0.0) | 0 (0.0) | 0 (0.0) | 0 (0.0) |
| F. My friends, colleagues | 7 (0.4) | 3 (0.5) | 4 (0.3) | 2 (0.4) | 1 (0.2) | 4 (0.6) | 0 (0.0) |

Results are presented as *n (%)*. Q1 against gender *p*=0.936, Q1 against year of study *p*<0.001, Q2 against gender *p*=0.112, Q2 against year of study *p*<0.001, Q3 against gender *p*=0.110, Q3 against year of study *p<0.001*, *p* for Fisher’s exact test. JD – junior doctors.

**Table 3.** Knowledge and practices regarding vaccination boosters in Q4 and Q25.

| **Question**  Answer | **Total (N=1821)** | **Gender** | | | **Year of study** | | | |
| --- | --- | --- | --- | --- | --- | --- | --- | --- |
|  |  | **Male (N=555)** | **Female (N=1259)** | | **1-2**  **(N=467)** | **3-4 (N=645)** | **5-6 (N=651)** | **JD (N=58)** |
| **Q4. Did you get vaccinated as part of the vaccination program of your country?** | | | | | | | | |
| A. Yes | 1765 (96.9) | 537 (96.8) | 1221 (97.0) | | 438 (93.8) | 629 (97.5) | 640 (98.3) | 58 (100) |
| B. No | 25 (1.4) | 6 (1.1) | 19 (1.5) | | 13 (2.8) | 7 (1.1) | 5 (0.8) | 0 (0.0) |
| C. I don't know | 31 (1.7) | 12 (2.2) | 19 (1.5) | | 16 (3.4) | 9 (1.4) | 6 (0.9) | 0 (0.0) |
| **Q25. Do you know that in order to be protected properly you need to get revaccinated for several vaccines?** | | | | | | | | |
| A. Yes, I am aware it and doing it properly. | 1239 (68.0) | 375 (67.6) | 860 (68.3) | 279 (59.7) | | 420 (65.1) | 495 (76.0) | 45 (77.6) |
| B. Yes, I am aware of it, but I am not sure if I have full vaccination. | 568 (31.2) | 173 (31.2) | 392 (31.1) | 184 (39.4) | | 220 (34.1) | 151 (23.2) | 13 (22.4) |
| C. No, this is the first time I hear about that. | 12 (0.7) | 6 (1.1) | 6 (0.5) | 4 (0.9) | | 5 (0.8) | 3 (0.5) | 0 (0.0) |
| D. No, there is no need because vaccination is always life-long protection | 2 (0.1) | 1 (0.2) | 1 (0.1) | 0 (0.0) | | 0 (0.0) | 2 (0.3) | 0 (0.0) |

Results are presented as *n (%)*. Q4 against gender *p*=0.481, Q4 against year of study *p*=0.004, Q25 against gender *p*=0.385, Q25 against year of study *p*<0.001, *p* for Fisher’s exact test. JD – junior doctors.

**Table 4.** Vaccination counselling and attitudes towards vaccination programs in pregnancy in Q26 and Q27.

| **Question**  Answer | **Total (N=1821)** | **Gender** | | **Year of study** | | | |
| --- | --- | --- | --- | --- | --- | --- | --- |
|  |  | **Male (N=555)** | **Female (N=1259)** | **1-2**  **(N=467)** | **3-4 (N=645)** | **5-6 (N=651)** | **JD (N=58)** |
| **Q26. Do you advise your relatives, friends, colleagues etc. to get vaccinated?** | | | | | | | |
| A. Yes | 1629 (89.5) | 477 (85.9) | 1146 (91.0) | 366 (78.4) | 586 (90.9) | 622 (95.5) | 55 (94.8) |
| B. No | 52 (2.9) | 22 (4.0) | 30 (2.4) | 26 (5.6) | 11 (1.7) | 13 (2.0) | 2 (3.4) |
| C. Never thought about that | 140 (7.7) | 56 (10.1) | 83 (6.6) | 75 (16.1) | 48 (7.4) | 16 (2.5) | 1 (1.7) |
| **Q27. Do you think that a more specific vaccination program should be available to pregnant women (e.g. seasonal flu, mumps, rubella)?** | | | | | | | |
| A. Yes, because that way the fetus will be protected against inborn anomalies and fewer miscarriages will occur | 1507 (82.8) | 478 (86.1) | 1025 (81.4) | 358 (76.7) | 536 (83.1) | 565 (86.8) | 48 (82.8) |
| B. No, because a specific programme is not safe to a pregnant woman or the fetus | 194 (10.7) | 46 (8.3) | 145 (11.5) | 67 (14.3) | 70 (10.9) | 55 (8.4) | 2 (3.4) |
| C. No, because the vaccine is not effective for the pregnant woman or the fetus. | 12 (0.7) | 4 (0.7) | 8 (0.6) | 6 (1.3) | 3 (0.5) | 3 (0.5) | 0 (0.0) |
| D. No, because everyone should have the right to choose. | 108 (5.9) | 27 (4.9) | 81 (6.4) | 36 (7.7) | 36 (5.6) | 28 (4.3) | 8 (13.8) |

Results are presented as *n (%)*. Q26 against gender *p*=0.005, Q26 against year of study *p*<0.001, Q27 against gender p=0.085, Q27 against year of study *p<0.001*, *p* for Fisher’s exact test. JD – junior doctors.

**Table 5**. Attitudes towards seasonal influenza vaccination in Q28 and Q29.

| **Question**  Answer | **Total (N=1821)** | **Gender** | | **Year of study** | | | |
| --- | --- | --- | --- | --- | --- | --- | --- |
|  |  | **Male**  **(N=555)** | **Female**  **(N=1259)** | **1-2**  **(N=467)** | **3-4 (N=645)** | **5-6 (N=651)** | **JD**  **(N=58)** |
| **Q28: What is your opinion about the seasonal flu vaccine?** | | | | | | | |
| A. It is an almost 100% protection against seasonal flu. | 126 (6.9) | 54 (9.7) | 72 (5.7) | 32 (6.9) | 48 (7.4) | 45 (6.9) | 1 (1.7) |
| B. It is not useful because the seasonal flu virus mutates constantly and there is a different type every year. | 310 (17.0) | 94 (16.9) | 214 (17.0) | 120 (25.7) | 109 (16.9) | 75 (11.5) | 6 (10.3) |
| C. It won't necessary prevent you from contracting the seasonal flu, but the disease will be less serious. | 1379 (75.7) | 405 (73.0) | 969 (77.0) | 313 (67.0) | 486 (75.3) | 529 (81.3) | 51 (87.9) |
| D. Vaccines in general are not effective and the seasonal flu vaccine is not an exception. | 6 (0.3) | 2 (0.4) | 4 (0.3) | 2 (0.4) | 2 (0.3) | 2 (0.3) | 0 (0.0) |
| **Q29: How often do you get vaccinated against seasonal flu?** | | | | | | | |
| A. Every other season. | 73 (4.0) | 23 (4.1) | 50 (4.0) | 17 (3.6) | 21 (3.3) | 29 (4.5) | 6 (10.3) |
| B. Every season. | 329 (18.1) | 96 (17.3) | 232 (18.4) | 57 (12.2) | 88 (13.6) | 167 (25.7) | 17 (29.3) |
| C. I have never been vaccinated against seasonal flu. | 836 (45.9) | 234 (42.2) | 599 (47.6) | 233 (49.9) | 315 (48.8) | 271 (41.6) | 17 (29.3) |
| D. I haven only been vaccinated once. | 263 (14.4) | 80 (14.4) | 181 (14.4) | 69 (14.8) | 108 (16.7) | 81 (12.4) | 5 (8.6) |
| E. Not regularly. | 320 (17.6) | 122 (22.0) | 197 (15.6) | 91 (19.5) | 113 (17.5) | 103 (15.8) | 13 (22.4) |

Results are presented as *n (%)*. Q28 against gender *p*=0.020, Q28 against year of study *p<0.001*, Q29 against gender *p*=0.022, Q29 against year of study *p<0.001*, *p* for Fisher’s exact test. JD – junior doctors.

**Table 6.** Attitudes towards mandatory vaccination of medical staff and MS in Q30 and Q31.

| **Question**  Answer | **Total (N=1821)** | **Gender** | | **Year of study** | | | |
| --- | --- | --- | --- | --- | --- | --- | --- |
|  |  | **Male (N=555)** | **Female (N=1259)** | **1-2 (N=467)** | **3-4 (N=645)** | **5-6 (N=651)** | **JD (N=58)** |
| **Q30: Do you think that a vaccine against seasonal flu and hepatitis B should be mandatory for medical staff (attending doctors, nurses etc.)?** | | | | | | | |
| A. No, because everyone should have the right to choose. | 227 (12.5) | 63 (11.4) | 164 (13.0) | 75 (16.1) | 77 (11.9) | 65 (10.0) | 10 (17.2) |
| B. No, because those vaccines are not effective. | 21 (1.2) | 9 (1.6) | 12 (1.0) | 9 (1.9) | 5 (0.8) | 6 (0.9) | 1 (1.7) |
| C. No, because those vaccines are not safe. | 7 (0.4) | 3 (0.5) | 4 (0.3) | 3 (0.6) | 2 (0.3) | 2 (0.3) | 0 (0.0) |
| D. Yes, because medical staff has a greater chance to get infected and then spread the spread the virus. | 1566 (86.0) | 480 (86.5) | 1079 (85.7) | 380 (81.4) | 561 (87.0) | 578 (88.8) | 47 (81.0) |
| **Q31: Do you think that a vaccine against seasonal flu and hepatitis B should be mandatory for medical students?** | | | | | | | |
| A. No, because everyone should have the right to choose. | 287 (15.8) | 85 (15.3) | 202 (16.0) | 106 (22.7) | 96 (14.9) | 75 (11.5) | 10 (17.2) |
| B. No, because these vaccines are not safe. | 6 (0.3) | 2 (0.4) | 4 (0.3) | 3 (0.6) | 1 (0.2) | 2 (0.3) | 0 (0.0) |
| C. No, because those vaccines are not effective. | 22 (1.2) | 8 (1.4) | 14 (1.1) | 9 (1.9) | 6 (0.9) | 6 (0.9) | 1 (1.7) |
| D. Yes, because medical students rotate through different departments in a hospital and can spread the virus. | 1506 (82.7) | 460 (82.9) | 1039 (82.5) | 349 (74.7) | 542 (84.0) | 568 (87.3) | 47 (81.0) |

Results are presented as *n (%)*. Q30 against gender *p*=0.384, Q30 against year of study *p*=0.038, Q31 against gender *p*=0.881, Q31 against year of study *p<0.001*, *p* for Fisher’s exact test. JD – junior doctors.

**Table 7.** Self-reported coverage for 19 vaccines in country-specific vaccination programs declared by medical students and junior doctors - total sample and against gender.

| **Types of vaccinations** | **Vaccine** | **Total (N=1821)** | | | **Gender** | | | | | | ***p*** |
| --- | --- | --- | --- | --- | --- | --- | --- | --- | --- | --- | --- |
|  |  |  |  |  | **Male (N=555)** | | | **Female (N=1259)** | | |  |
|  |  | **Yes** | **No** | **Don’t know** | **Yes** | **No** | **Don’t know** | **Yes** | **No** | **Don’t know** |  |
| Childhood schedules in all EU/EEA | Tetanus | 94.2 | 3.0 | 2.7 | 93.9 | 3.1 | 3.1 | 94.4 | 3.0 | 2.6 | 0.848 |
|  | Diphtheria | 89.0 | 3.6 | 7.4 | 88.8 | 3.4 | 7.7 | 89.0 | 3.7 | 7.3 | 0.908 |
|  | Poliomyelitis | 85.7 | 4.9 | 9.4 | 85.4 | 4.5 | 10.1 | 85.9 | 5.1 | 9.1 | 0.704 |
|  | Rubella | 83.3 | 6.9 | 9.8 | 79.5 | 8.5 | 12.1 | 85.1 | 6.2 | 8.7 | 0.012 |
|  | Measles | 81.0 | 8.7 | 10.3 | 80.5 | 9.2 | 10.3 | 81.3 | 8.6 | 10.2 | 0.898 |
|  | Mumps | 80.2 | 8.6 | 11.2 | 77.8 | 9.5 | 12.6 | 81.3 | 8.3 | 10.5 | 0.229 |
|  | Pertussis | 79.3 | 8.0 | 12.7 | 79.5 | 8.1 | 12.4 | 79.2 | 7.9 | 12.9 | 0.963 |
|  | *Haemophilus influenzae* type b | 53.8 | 25.7 | 20.5 | 54.2 | 23.6 | 22.2 | 53.7 | 26.6 | 19.7 | 0.287 |
|  | Human papillomavirus | 25.0 | 65.4 | 9.6 | 7.2 | 78.4 | 14.4 | 33.0 | 59.7 | 7.4 | <0.001 |
| Additional childhood  vaccines | Tuberculosis | 60.6 | 29.8 | 9.6 | 57.1 | 31.7 | 11.2 | 62.2 | 29.0 | 8.8 | 0.088 |
|  | Hepatitis A | 40.5 | 47.4 | 12.0 | 42.9 | 43.4 | 13.7 | 39.7 | 49.1 | 11.2 | 0.058 |
|  | Meningococci | 32.2 | 46.8 | 20.9 | 33.2 | 42.0 | 24.9 | 32.0 | 48.8 | 19.1 | 0.006 |
|  | Pneumococci | 25.6 | 51.8 | 22.6 | 26.7 | 49.9 | 23.4 | 25.2 | 52.6 | 22.2 | 0.573 |
|  | Chickenpox | 24.6 | 60.6 | 14.8 | 23.2 | 60.2 | 16.6 | 25.3 | 60.8 | 13.9 | 0.281 |
| Vaccines recommended both for children and for adults in risk groups | Hepatitis B | 83.4 | 11.3 | 5.3 | 78.9 | 13.3 | 7.7 | 85.5 | 10.2 | 4.3 | 0.001 |
|  | Influenza/seasonal flu | 32.9 | 62.4 | 4.6 | 34.2 | 58.4 | 7.4 | 32.4 | 64.2 | 3.4 | 0.001 |
| Vaccines for adults | Typhus | 21.1 | 56.8 | 22.1 | 20.2 | 54.1 | 25.8 | 21.6 | 58.0 | 20.4 | 0.041 |
|  | Cholera | 12.9 | 67.4 | 19.7 | 14.6 | 62.9 | 22.5 | 12.1 | 69.5 | 18.4 | 0.020 |
|  | Smallpox | 24.4 | 53.7 | 22.0 | 25.0 | 50.6 | 24.3 | 24.1 | 55.0 | 20.9 | 0.160 |

Results are presented as *%*. Yes + No + Don’t know =100% for every vaccine in every group of respondents. *p* for Fisher’s exact test**.**

**Table 8.** Self-reported coverage for 19 vaccines in country-specific vaccination programs declared by medical students and junior doctors – against year of study.

| **Types of vaccinations** | **Vaccine** | **Year of study** | | | | | | | | | | | | ***p*** |
| --- | --- | --- | --- | --- | --- | --- | --- | --- | --- | --- | --- | --- | --- | --- |
|  |  | **1-2 (N=467)** | | | **3-4 (N=645)** | | | **5-6 (N=651)** | | | **JD (N=58)** | | |  |
|  |  | **Yes** | **No** | **Don’t know** | **Yes** | **No** | **Don’t know** | **Yes** | **No** | **Don’t know** | **Yes** | **No** | **Don’t know** |  |
| Childhood schedules in all EU/EEA | Tetanus | 88.0 | 6.6 | 5.4 | 94.7 | 2.5 | 2.8 | 97.7 | 1.2 | 1.1 | 100.0 | 0.0 | 0.0 | <0.001 |
|  | Diphtheria | 73.4 | 8.1 | 18.4 | 91.2 | 2.5 | 6.4 | 97.2 | 1.5 | 1.2 | 96.6 | 3.4 | 0.0 | <0.001 |
|  | Poliomyelitis | 71.7 | 8.1 | 20.1 | 85.3 | 5.1 | 9.6 | 94.9 | 2.8 | 2.3 | 100.0 | 0.0 | 0.0 | <0.001 |
|  | Rubella | 67.2 | 10.1 | 22.7 | 85.4 | 6.7 | 7.9 | 92.3 | 4.5 | 3.2 | 87.9 | 10.3 | 1.7 | <0.001 |
|  | Measles | 68.7 | 12.8 | 18.4 | 80.8 | 8.1 | 11.2 | 89.4 | 6.3 | 4.3 | 87.9 | 10.3 | 1.7 | <0.001 |
|  | Mumps | 65.7 | 12.6 | 21.6 | 80.0 | 8.2 | 11.8 | 90.3 | 5.7 | 4.0 | 84.5 | 13.8 | 1.7 | <0.001 |
|  | Pertussis | 55.5 | 14.8 | 29.8 | 82.3 | 6.4 | 11.3 | 92.6 | 4.5 | 2.9 | 87.9 | 10.3 | 1.7 | <0.001 |
|  | *Haemophilus influenzae* type b | 40.3 | 25.9 | 33.8 | 55.8 | 23.4 | 20.8 | 61.9 | 26.4 | 11.7 | 50.0 | 41.4 | 8.6 | <0.001 |
|  | Human papillomavirus | 30.4 | 51.0 | 18.6 | 27.6 | 61.7 | 10.7 | 19.0 | 78.2 | 2.8 | 20.7 | 79.3 | 0.0 | <0.001 |
| Additional childhood  vaccines | Tuberculosis | 49.0 | 33.2 | 17.8 | 58.1 | 31.0 | 10.9 | 70.7 | 26.1 | 3.2 | 69.0 | 31.0 | 0.0 | <0.001 |
|  | Hepatitis A | 52.2 | 27.8 | 19.9 | 40.6 | 47.3 | 12.1 | 33.0 | 59.9 | 7.1 | 29.3 | 67.2 | 3.4 | <0.001 |
|  | Meningococci | 38.5 | 28.7 | 32.8 | 31.0 | 44.2 | 24.8 | 29.3 | 60.2 | 10.4 | 27.6 | 72.4 | 0.0 | <0.001 |
|  | Pneumococci | 32.5 | 31.3 | 36.2 | 26.5 | 46.5 | 27.0 | 20.0 | 69.6 | 10.4 | 22.4 | 75.9 | 1.7 | <0.001 |
|  | Chickenpox | 26.3 | 51.2 | 22.5 | 27.0 | 56.6 | 16.4 | 20.4 | 70.8 | 8.8 | 31.0 | 67.2 | 1.7 | <0.001 |
| Vaccines recommended both for children and for adults in risk groups | Hepatitis B | 79.2 | 10.1 | 10.7 | 84.2 | 10.5 | 5.3 | 85.6 | 12.7 | 1.7 | 84.5 | 12.1 | 3.4 | <0.001 |
|  | Influenza/seasonal flu | 33.6 | 58.0 | 8.4 | 28.4 | 67.6 | 4.0 | 35.6 | 61.4 | 2.9 | 48.3 | 51.7 | 0.0 | <0.001 |
| Vaccines for adults | Typhus | 24.2 | 38.3 | 37.5 | 21.9 | 53.6 | 24.5 | 18.6 | 71.0 | 10.4 | 17.2 | 81.0 | 1.7 | <0.001 |
|  | Cholera | 18.2 | 48.2 | 33.6 | 14.4 | 64.3 | 21.2 | 8.1 | 82.0 | 9.8 | 5.2 | 93.1 | 1.7 | <0.001 |
|  | Smallpox | 29.3 | 38.3 | 32.3 | 26.0 | 48.2 | 25.7 | 19.7 | 67.9 | 12.4 | 19.0 | 77.6 | 3.4 | <0.001 |

Results are presented as *%*. Yes + No + Don’t know =100% for every vaccine in every group of respondents. *p* for Fisher’s exact test**.** JD – junior doctors
